# Increased Incidence of Benign Pancreatic Pathology following Pancreaticoduodenectomy for Presumed Malignancy over 10 Years despite Increased Use of Endoscopic Ultrasound

**DOI:** 10.1155/2014/701535

**Published:** 2014-06-05

**Authors:** Shadi S. Yarandi, Thomas Runge, Lei Wang, Zhijian Liu, Yueping Jiang, Saurabh Chawla, Kevin E. Woods, Steven Keilin, Field F. Willingham, Hong Xu, Qiang Cai

**Affiliations:** Division of Digestive Diseases, Emory University School of Medicine, 1365 Clifton Road, B1262, Atlanta, GA 30322, USA

## Abstract

Despite using imaging studies, tissue sampling, and serologic tests about 5–10% of surgeries done for presumed pancreatic malignancies will have benign findings on final pathology. Endoscopic ultrasound (EUS) is used with increasing frequency to study pancreatic masses. The aim of this study is to examine the effect of EUS on prevalence of benign diseases undergoing Whipple over the last decade. Patients who underwent Whipple procedure for presumed malignancy at Emory University Hospital from 1998 to 2011 were selected. Demographic data, history of smoking and drinking, history of diabetes and pancreatitis, imaging data, pathology reports, and tumor markers were extracted. 878 patients were found. 95 (10.82%) patients had benign disease. Prevalence of benign finding had increased over the recent years despite using more EUS. Logistic regression models showed that abdominal pain (OR: 5.829, 95% CI 2.681–12.674, *P* ≤ 0.001) and alcohol abuse (OR: 3.221, CI 95%: 1.362–7.261, *P*: 0.002) were predictors of benign diseases. Jaundice (OR: 0.221, 95% CI: 0.084–0.58, *P*: 0.002), mass (OR: 0.145, 95% CI: 0.043–0.485, *P*: 0.008), and ductal dilation (OR: 0.297, 95% CI 0.134–0.657, *P*: 0.003) were associated with malignancy. Use of imaging studies, ERCP, and EUS has not decreased the percentage of benign findings after surgery for presumed pancreatic malignancy.

## 1. Introduction

Pancreatic cancer accounts for 2% of newly diagnosed malignancies, with pancreaticoduodenectomy (The Whipple procedure) being the only potentially curative treatment [1]. Differentiating between pancreatic carcinoma and benign diseases of the pancreas such as chronic pancreatitis is challenging. Pancreatic cancer can present with vague symptoms that overlap with the symptomatology of benign diseases and have an insidious course. The imaging findings also overlap between benign and malignant diseases and no single finding such as pancreatic duct dilation, focal mass, cyst, or abnormal enhancement pattern can reliably make the differentiation. In addition, secondary inflammatory changes are often seen in pancreatic cancer while chronic pancreatitis is associated with an increased risk of pancreatic carcinoma [2].

Although the outcome of the Whipple procedure has improved significantly over the past years and is associated with 1-2% mortality when performed in large volume centers, the morbidity of the procedure remains high [3]. Therefore, constant efforts are being made to improve available diagnostic tools in order to prevent performing Whipple for benign diseases. Despite the advances in imaging techniques, 5–10% of patients who undergo Whipple procedure for a presumed malignant disease will have benign pathology on the final pathologic review [4–12]. Usually a combination of computed tomography (CT), magnetic resonance imaging/magnetic resonance cholangiopancreatography (MRI/MRCP), endoscopic retrograde cholangiopancreatography (ERCP) with brushing, endoscopic ultrasound/fine needle aspiration (EUS/FNA), and serology testing is performed before the decision for surgery is made. All of these methods have false negative results and relatively low negative predictive values, adding uncertainty to the decision making process. Given the high mortality of untreated pancreatic cancer, surgeons often decide to offer surgery to the patients in whom a malignant lesion is clinically suspected even if the results of tissue sampling are unconvincing for malignancy.

In the current study, we have reviewed Whipple procedures that have been performed for presumed malignant pancreatic disease over the past decade at Emory University Hospital. The aim of this study is to examine the effect of presurgical tissue sampling by EUS/FNA on the rate of finding benign pathology from Whipple procedure performed for presumed malignant disease. We also performed a multivariate binary logistic regression analysis to investigate if any of the collected data can help differentiating benign disease from malignant before surgery.

## 2. Patients and Methods

With approval of institutional review board at Emory University, the admitting and discharge diagnosis and procedure codes were queried from the electronic medical records at Emory University Hospital (EUH). The current procedural terminology (CPT) and International Classification of Diseases Ninth Revision (ICD-9) codes for “pancreaticoduodenectomy” were used to identify patients who underwent the surgery.

A retrospective review from January 1998 to December 2011 of the patients undergoing Whipple procedure for presumed malignant pancreatic disease at Emory University Hospital was conducted. Patients with a diagnosis of chronic pancreatitis undergoing resection for relief of the symptoms were excluded from the analysis. A total of 872 patients were identified. Electronic medical records were reviewed and the following data were collected: demographics, clinical presentation including weight loss (10% or more of body weight), jaundice, abdominal, pain, acute or chronic pancreatitis, diabetes mellitus, history of smoking, history of alcohol over use, radiographic findings including CT scan, MRI/MRCP, ERCP, EUS, diagnostic preoperative pathologic data including endoscopic and image-guided biopsies, and final pathologic report after surgery.

There was no universal preoperative imaging protocol over years and imaging studies were performed based on the evaluation of the case by the attending surgeons or the consulting gastroenterologists. CT scans were often performed before referral to our center and findings were documented in the surgeons note in all of the cases. MRI/MRCP, ERCP, and EUS/FNA were performed at EUH and the records of radiology and pathology reports in case of biopsy were reviewed. Preoperative specimens with conclusive evidence of malignancy or those suspicious for malignancy, showing dysplasia or evidence of neoplastic processes, were considered positive. Biopsies described as inflammatory, reactive, or atypical were considered nondiagnostic. All specimens were reviewed by experienced gastrointestinal pathologists at EUH. Patients with benign disease were characterized based on the histopathological findings. Chronic fibrosing pancreatitis was defined by pathologic findings including inflammatory changes, fat necrosis, fibrosis, and loss of acinar cells.

All continuous data were represented as mean +/− standard error of the mean. Frequency data was used otherwise. Binary logistic regression models were constructed to assess the potential predictor factors of benign disease in patients undergoing Whipple procedure.

## 3. Results

**Figure 1:**
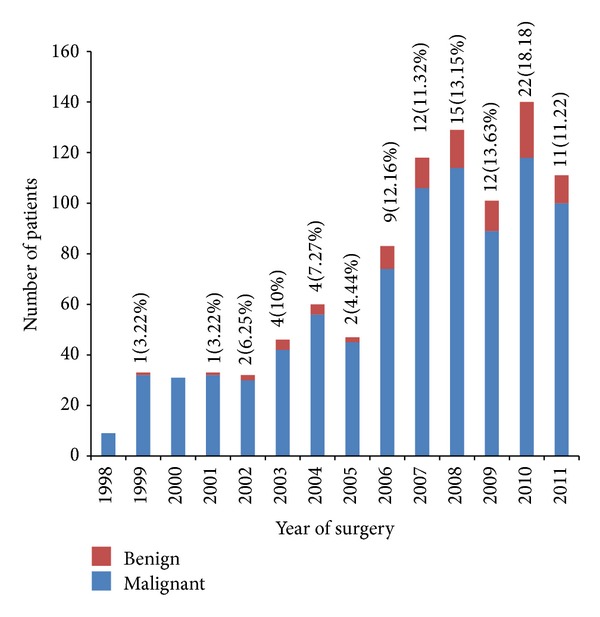
Distribution of number of surgeries and percentage of benign pathologic findings per year. Number of Whipple surgeries performed each year for presumed malignant disease at EUH from 1998 to 2011. Number and percentage of benign findings are listed above each bar. As illustrated in the figure, total number of surgeries and percentage of benign findings have been increased over the years.

From 878 Whipple procedures performed between January 1998 and December 2011, 95 (10.8%) patients had benign pathology on the final pathology report. Number of benign findings as well as the percentage of benign finding to total number of operation performed each year has been increased over the years despite increased number of EUS/FNA performed in each year (Figure 1).

Out of 95 patients with benign findings, 43 (45.3%) were female and 52 (54.7%) were male. Age of the patients with benign findings ranges between 20 and 81 with mean of 53.66 (standard deviation: 11.54 years). 65 (68.4%) patients were Caucasian while 28 (29.5) were African American, 1 Hispanic, and 1 Asian. 57 (60%) patients had history of smoking and 42 (44.2%) had history of excessive alcohol use. Ten patients had history of DM (10.5%) and 40 (42.5%) had history of at least one episode of pancreatitis. Summary of this data is presented in Table 1.

**Figure 2:**
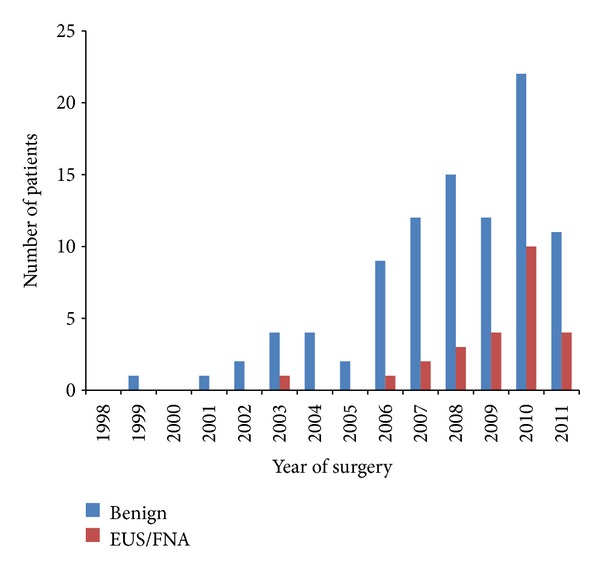
Number of EUS/FNA performed between 1998 and 2011 in patients with benign disease who underwent Whipple procedure for presumed malignancy. Number of EUS/FNA in patients with benign findings after Whipple procedure performed each year for presumed malignant disease at EUH from 1998 to 2011. As illustrated, number of EUS/FNA performed has increased over the years, but has not led to decreased number of Whipple procedures performed for benign diseases.

The most common complaint at the time of presentation among patients with benign finding was abdominal pain which was reported by 91 (95.8%) patients. 69 (72.6%) patients in benign group also reported some degree of weight loss. Jaundice that was reported to be more common in malignant pathology in some of the previous studies was reported by 25 (26.3%) patients with benign disease. As for imaging studies that were performed before surgery, 49 patients in benign group had CT scan at EUH and 46 had CT scan done before referral to EUH with reports available to review. MRI with or without MRCP was done in 85 (89.5%) of patients with benign pathology. EUS with FNA aspiration was done in 25 (26.3%) and ERCP with brushing was done in 32 (33.7%) of patients with benign pathology. Results of these tests were invariably nondiagnostic, leading the surgeon to pursue the surgery. Number of EUS/FNA and ERCPs done before surgery had increased over the years, as illustrated in Figure 2. Among 783 patients with malignant disease on final pathology, 274 (35%) had underwent ERCP and 92 (11.7%) had underwent EUS/FNA before the surgery. The same trend toward more EUS/FNAs over recent years was observed in patients with malignant disease as well. For example, 25 (27% of the total number of the EUS procedures) EUS/FNA was performed in 2011 and 22 was performed (23.9%) in 2010 alone.

Among these 95 patients, imaging studies including CT and MRI reported a solid mass within the head of pancreas in 56 (58.9%) of patients with the average size of 13.7 millimeter and standard deviation of 15.45 millimeter. 23 (24.2) patients were reported to have a cystic mass or cyst at the head of pancreas with the average size of 7.6 mm and standard deviation of 16.48 mm. An abnormal pattern of enhancement of the mass, or periductal enhancement without a mass, was reported in 62 (65.3%) patients. Abnormal enhancement was reported with multiple patterns including increased early enhancement, increased delayed enhancement, or decreased enhancement (Table 2). In the imaging studies of 32 (33.7%) patients there was evidence of inflammation; abnormally enlarged abdominal lymph nodes were reported in imaging of 6 (6.3%) patients; imaging studies of 62 (65.3%) patients showed some degree of pancreatic duct dilation and invasion or pressure to vascular structures including the portal vein or the superior mesenteric artery was seen in 10 (10.5%) patients.

The final histologic findings of these patients are listed in Table 3. The most common findings were chronic fibrosing pancreatitis with or without focal mass like fibrosis, paraduodenal pancreatitis, chronic pancreatitis, and focal pancreatitis. There were 6 cases of lymphoplasmacytic sclerosing autoimmune pancreatitis. Rare conditions such as arteriovenous malformation (AVM), thrombosed aneurism, xanthogranulomatosis chronic inflammation, and lipoma were reported in few cases.

Binary logical regression models were constructed using gender, race, smoking, alcohol abuse, chronic pancreatitis, presenting symptoms including abdominal pain, jaundice and weight loss, radiographic findings including presence of mass, ductal dilation, lymphadenopathy, and vascular invasion as independent predictors. This analysis showed that Odds ratio of benign disease in patients with abdominal pain (OR: 5.829, 95% CI 2.681–12.674, *P* ≤ 0.001) as the main presenting symptom and alcohol abuse (OR: 3.221, CI 95%: 1.362–7.261, *P*: 0.002) was significantly higher compared to the patients with pancreatic cancer. On the other hand, OR of benign disease in patients presenting with jaundice (OR: 0.221, 95% CI: 0.084–0.58, *P* value: 0.002), with a mass present in imaging (OR: 0.145, 95% CI: 0.043–0.485, *P*: 0.008) and with ductal dilation (OR: 0.297, 95% CI 0.134–0.657, *P*: 0.003), was significantly lower compared to the malignant disease (Table 4).

## 4. Discussion

In this study, out of 878 Whipple procedures performed on the patients with presumed pancreatic cancer over ten years, the percentage of benign finding was 10.8 with a trend toward increase over recent years despite increased number of EUS/FNA performed. The most common pathologic finding was chronic fibrosing pancreatitis, most likely in the setting of chronic pancreatitis secondary to alcohol abuse. Also, the results of this study showed that abdominal pain as main presenting symptom and a history of alcohol abuse is associated with benign pancreatic diseases while jaundice, presence of a mass in imaging studies, and bile duct/pancreatic duct dilation are associated with malignancies.

Previously, 11 academic institutions have published their experience with Whipple procedure and prevalence of unexpected benign findings. Thompson et al. initially reported 67 patients who underwent surgery between 1978 and 1993 at the University of Nebraska and reported 11 patients with benign findings [10]. They later published a follow-up report of 132 patients who underwent Whipple procedure between 1995 and 2008, with the prevalence of benign findings reported as 12.9%, most commonly chronic fibrosing pancreatitis. In this follow-up report, similar to our finding, incidence of benign pathology was increased over years [7]. They attributed this finding to decreased use of EUS in their center, however in our center EUS or ERCP was performed more frequently during the recent years.

Tessler et al. reported their experience at Henry Ford Medical Center between 1998 and 2004. Among their patients, 102 patients had no tissue diagnosis before the surgery and 27 ended up having benign pathology [9]. Large number of patients in their series had EUS (*n* = 80) or ERCP (*n* = 68) before the surgery. Despite the fact that they used EUS and ERCP frequently, the percentage of benign findings was similar to the other reports. They tried to combine several imaging, laboratory, and symptomatology findings to develop a scoring system that can predict malignant versus benign disease. They suggested that in the appropriate clinical setting, a combination of weight loss, jaundice, and increased CA 19-9, often in combination with a biliary stricture or pancreatic mass should be strongly considered for surgery even if a preoperative tissue diagnosis is not present [8]. However, this criterion has not been validated by other studies.

Barnes et al. reviewed 510 pancreaticoduodenectomies performed at The Johns Hopkins Hospital during an 8-year period. One hundred and eight patients (21%) underwent surgery for benign disease, 83 (16%) of those underwent surgery for suspicion of malignancy [5]. In another report from the Johns Hopkins Hospital, Abraham et al. published a retrospective review of 442 patients who underwent the procedure between 1999 and 2001, in whom 40 patients (9.2%) were found to have some form of benign inflammatory condition of the pancreas or biliary tract [4]. van Gulik et al. described 220 patients who underwent Whipple and reported 6% benign findings. They suggested that at least 5% of benign finding is expected when performing Whipple procedure for a suspected malignant disease and given the grim prognosis of the pancreatic cancer this should not stop surgeons from performing the procedure on patients with clinically suspected malignancy but with no other confirming data [11].

In a report from Mayo Clinic, Smith et al. reviewed 484 patients who underwent Whipple procedure for suspected periampullary malignancy and found chronic inflammatory disease on final pathologic assessment in 24 patients (5%) [8]. Weber et al. reported a 4.5% incidence of pancreatitis on final pathologic review in 1,287 patients undergoing Whipple procedure at Memorial-Sloan Kettering [12]. Northwestern experience was published by Kennedy et al. They reported 162 patients undergoing Whipple surgery between 1993 and 2004, among them 21 patient (12.9%) had benign findings. In their experience, jaundice was more common in patients with malignant pathology but they were not able to find any other significant difference between patients with malignant or benign disease [13]. In their study, CT, MRI, and EUS suggested the presence of a discrete mass lesion in 67%, 71%, and 67% of patients, respectively, and they concluded that these findings confirm previous reports suggesting that these imaging tests can be highly accurate in predicting resectable versus unresectable disease in periampullary cancer but are less reliable in the differential diagnosis of pancreatic cancer and chronic pancreatitis [14].

In a series of more than 400 patients operated on for presumed pancreatic cancer, Patlas and colleagues [15] observed that 21 patients had pancreatitis and one had tuberculosis on final pathology or by clinical follow-up studies. All 22 patients were presented with painless jaundice and underwent CT scans and transabdominal ultrasounds, 18 had ERCP, and 9 had MRI. Ten had fine needle biopsies with no biopsy showing malignancy. A report from Duke University reported 494 patients who underwent Whipple procedure for suspected malignancy between 1992 and 2007 and showed that despite the use of aggressive preoperative work-up for pancreatic cancer, up to 7% of patients that undergo resection will have benign disease on postoperative pathologic examination. All of these patients had findings concerning for malignancy on both CT scanning and endosonography preoperatively [16]. In 2 more recent European studies, prevalence of benign disease in patients who underwent pancreatoduodenectomy for presumed malignancy reported to be 8.4% [17] and 15.6% [6].

In our report, despite the increasing use of EUS with FNA and ERCP with brushing, number of surgeries for benign pathology has been increased over the years. In these patients, findings of brush cytology and FNA have been invariably benign or nondiagnostic, but given the low negative predictive value of benign finding presence of mass on MRI/EUS, elevated CA 19-9, and high clinical suspicion for malignancy Whipple procedure was performed regardless of presurgical tissue diagnosis. One possible explanation for increased number of surgeries over time is that as the experience of surgeons with Whipple procedure has increased over the time and the morbidity from the procedure has decreased, more patients with negative work-up but high clinical suspicious have been selected for surgery.

Despite addition of EUS/FNA for examination of pancreatic masses, a highly sensitive and specific criteria for selecting patients for Whipple based on imaging, presurgical tissue pathology, and serologic data is still lacking and until such criteria is developed, about 10% of surgeries will be done for benign diseases that are being presumed malignant based on clinical judgment in combination with a spectrum of imaging findings. Given the lack of any definitive test to separate malignant from benign disease, this approach is reasonable since Whipple procedure is the only chance of increasing survival in pancreatic cancer. Further studies are required to formulate practical criteria to diagnose benign disease before surgery.

## Figures and Tables

**Table 1:** Demographic data of patients with benign disease.

Category	Number (percent)
Gender	
Male	52 (54.7)
Female	43 (45.3)
Race	
White	65 (68.4)
African American	28 (29.5)
Asian	1 (1.1)
Hispanic	1 (1.1)
Past medical history	
Diabetes	10 (10.5)
Pancreatitis	40 (42.1)
Social history	
Smoking	57 (60)
Alcohol	42 (44.2)
Presenting symptom	
Abdominal pain	91 (95.8)
Jaundice	25 (26.3)
Weight loss	69 (72.6)

**Table 2:** Imaging modalities and findings of patients with benign disease.

Category	Number (percent)
Imaging type	
MRI	85 (89.5)
EUS	25 (26.3)
ERCP	32 (33.7)
Space occupying lesion	
Mass	56 (58.9)
Size	13.71 (15.43 SD)
Cyst	23 (24.2)
Size	7.6 (16.48)
Abnormal enhancement	
Periductal	7 (7.36)
Decreased enhancement	13 (13.68)
Increased early enhancement	24 (25.6)
Increased delayed enhancement	18 (18.94)
Other	
Inflammation	32 (33.7)
Duct dilation	62 (65.3)
Lymph node	6 (6.3)
Vascular involvement	10 (10.5)

**Table 3:** Frequency of Benign Findings.

Type	Number (percent)
PLSP autoimmune pancreatitis	6 (6.3)
Chronic fibrosing pancreatitis	20 (21)
Chronic pancreatitis	18 (18.94)
Focal active pancreatitis	11 (11.57)
Perideudonal pancreatitis	14 (14.73)
AVM	1 (1)
Chronic pancreatitis with infection	2 (2.1)
Granulomatose pancreatitis	1 (1)
Necrotizing pancreatitis	5 (5.26)
Pseudotumoral pancreatitis	6 (6.3)
Focal fat necrosis	2 (2.1)
Chronic pancreatitis with ductal stone	2 (2.1)
Lipoma	1 (1)
Lipomatose pseudohypertrophy	1 (1)
Intraductal adenoma	1 (1)
Xanthogranulomatous inflammation	1 (1)
Thrombosed pseudoaneurysm	1 (1)
Chronic pancreatitis with thrombosed vessels	1 (1)
Chronic pancreatitis due to deudonal diverticulum	1 (1)

**Table 4:** Logistic regression models for benign findings in patients undergoing Whipple procedure for presumed malignancy.

Variable	OR	95% CI	*P* value
Gender (female)	1.229	0.625–2.49	0.55
Race (African American)	0.804	0.390–1.66	0.55
Smoking	0.966	0.445–2.09	0.93
Chronic pancreatitis	1.072	0.184–1.168	0.102
Alcohol abuse	3.221	1.362–7.261	0.002
Abdominal pain	5.829	2.681–12.674	<0.001
Jaundice	0.221	0.084–0.58	0.002
Weight loss	1.566	0.94–1.89	0.078
Mass	0.145	0.043–0.485	0.008
Dilation of ducts	0.297	0.134–0.657	0.003
Lymphadenopathy	0.874	0.478–4.736	0.485
Vascular invasion	1.00	0.357–2.80	0.99
